# Etymologia: Buruli Ulcer

**DOI:** 10.3201/eid2603.ET2603

**Published:** 2020-03

**Authors:** Ronnie Henry

**Keywords:** Buruli ulcer, Mycobacterium ulcerans, bacteria, tuberculosis and other mycobacteria, skin ulcers, cutaneous infections, James Augustus Grant, Peter MacCallum, Uganda

## Buruli ulcer [booʹrǝ-le ulʹsǝr]

Named for Buruli County (now Nakasongola District), Uganda, where large numbers of cases were reported in the 1960s, Buruli ulcer (from the Latin *ulcus*, “sore”) is a cutaneous infection with *Mycobacterium ulcerans*. This bacterium produces a unique toxin (mycolactone), which causes rapid and extensive skin ulceration that is relatively painless. Buruli ulcer was first described by Sir Albert Cook in 1897. However, in his book *A Walk Across Africa*, describing his participation in the 1860 expedition to find the source of the Nile River, Scottish explorer James Augustus Grant might have earlier described Buruli ulcer (Figure):

**Figure:**
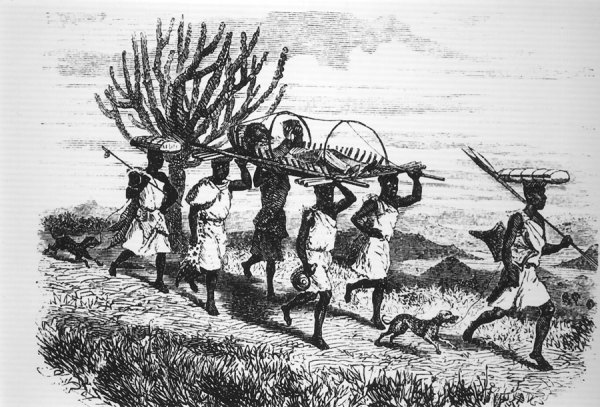
Captain Grant leaving Karague, carried on a wicker stretcher. Note his bent right leg. (From *Journal of the Discovery of the Source of the Nile*, by John Hanning Speke, 1863, p. 401.) Source: Wikipedia, https://en.wikipedia.org/wiki/File:James_Augustus_Grant.jpg

“The right leg, from above the knee, became deformed with inflammation, and remained for a month in this unaccountable state, giving intense pain, which was relieved temporarily by a deep incision and copious discharge. For three months, fresh abscesses formed, and other incisions were made; my strength was prostrated; the knee stiff and alarmingly bent, and walking was impracticable.”

Australian physician Peter MacCallum identified the causative organism in 1948. More than 33 countries in Africa, Central and South America, and the Western Pacific report cases of Buruli ulcer. Transmission is not well understood, which hampers the ability to prevent infection. Buruli ulcer is considered a public health problem in West Africa, and rates are also high in Victoria, Australia.
